# Effects of different dietary n-6/n-3 polyunsaturated fatty acid ratios on boar reproduction

**DOI:** 10.1186/s12944-016-0193-8

**Published:** 2016-02-16

**Authors:** Yan Lin, Xu Cheng, Jiude Mao, De Wu, Bo Ren, Sheng-Yu Xu, Zheng-Feng Fang, Lian-Qiang Che, Cai-Mei Wu, Jian Li

**Affiliations:** Key Laboratory for Animal Disease Resistance Nutrition of the Ministry of Education of China, Institute of Animal Nutrition, Sichuan Agricultural University, Ya’an, 625001 China; Division of Animal Sciences, University of Missouri, Columbia, MO 65211 USA

**Keywords:** n-3 fatty acids, n-6 fatty acids, Ratio, Boar, Reproduction

## Abstract

**Background:**

N-3 and N-6 polyunsaturated fatty acids are widely used in reproduction, yet few studies have addressed the effects of dietary n-6/n-3 ratios on boar reproduction. The present study aimed to determine the effects of different dietary n-6/n-3 ratios on the reproductive performance ofn breeding boars. Thirty-two boars with body weights of 15.0 ± 1.4 kg were divided into four treatments (C, T1, T2, T3) and fed diets with different n-6/n-3 fatty acid ratios (29.06:1, 20.07:1, 1:1, 1:17.96, respectively) for 174 days.

**Results:**

The highest testis index was observed for treatment T2. Sperm density and total sperm number per ejaculate in the T2 treatment were significantly higher than those in all other treatments, whereas the sperm deformity rate was the lowest. Interestingly, the fatty acid compositions and ratios of sperm were consistent with dietary treatments. Acid phosphatase and fructose concentration of seminal plasma, and the total superoxide dismutase and glutathione peroxidase of sperm in T2 were higher than those in other treatments. The concentration of testosterone and prostaglandin E2 increased in boars fed on diets supplemented with fatty acids as compared with boars subjected to the C grouptreatment, reaching a peak at n-6/n-3 fatty acid ratios of 1:1. Furthermore, higher expression of Δ^6^-fatty acid desaturase and peroxisome proliferator activated receptor-α in spermatozoa of the T2 treatment were observed, indicating more vigorous metabolism and intensive hormonal regulation.

**Conclusions:**

Our data suggest that the ideal n-6/n-3 ratio in the diet of breeding boars is 1:1, and proper balancing of n-6/n-3 fatty acids plays an important role in male reproduction.

## Background

Previous reports have revealed that the fatty acid content of spermatozoa in bulls, boars, rabbits, and humans are very high and has a unique function [1]. Intake of different types and sources of polyunsaturated fatty acids (PUFA) has been shown to change the fatty acid composition of animal sperm and affect sperm quality [2–4]. In fact, researchers have discovered that the male sperm quality can be affected by the pathway of PUFA metabolism [5], oxidative stress [6], hormone levels, and physiological function of the epididymis [7, 8].

The few studies published to-date regarding the effects of PUFA on human and animal male reproduction have reported divergent findings. Fish oil (rich in n-3 PUFA) has been shown to alter sperm structure and penetration resistance, and to increase sperm number [9] and antioxidant capacity [10]. However, some studies report no effect of fish oil on sperm quality [11, 12]. Mohammad et al. [13, 14] reported that the consumption of unsaturated fatty acids improved sperm antioxidant capacity of infertile men. Corn oil (rich in n-6 PUFA) and Vitamin E supplements increased the semen volume and sperm antioxidant properties [15]. Different oils have different fatty acid compositions, and we hypothesize that the reason for the above noted inconsistencies may be different dietary n-6/n-3 PUFA ratios; assuming no differences in fatty acid level. However, relevant information on the effects of PUFA ratios on male animals is rare. Castellano et al. [16] found that the fatty acid composition of boar testis changed in a variety of phospholipids hat after fish oil supplement, and the ratio of n-6/n-3 PUFA in sphingomyelin was reduced. On the other hand, adding tuna oil to the diet of the boars changed the fatty acid composition of sperm and seminal plasma, and the n-6/n-3 ratios in sperm and seminal plasma were reported as 2.26:1 and 0.997:1, respectively [17]. Furthermore, we found that sperm density, vitality and sperm morphological integrity in rats, were greatly improved when the dietary n-6/n-3 ratio of PUFA was 1.52:1 [18]. Thus, we hypothesized that different proportions of PUFA would affect the reproductive performance of boars. The objective of the present study was to determine the effects of different ratios of n-6/n-3 PUFA on testis development and reproductive performance in breeding boars.

## Methods

### Experimental design

All experimental protocols were approved by the Biosafety and Animal Care and Use Committees at Sichuan Agriculture University. Thirty-two boars with an average body weight of 15.0 ± 1.4 kg were randomly assigned to 4 groups: 3 treatment groups (T1, T2 and T3) fed with different ratios of dietary sunflower oil and fish oil, and a control group (C). Diets were formulated based on NRC recommendations (2012) [19] for breeding boars according to body weight. The fatty acid compositions of the oils and the basal diet (C, without oil supplementation) are presented in Table 1, and the fatty acid compositions of all diets are presented in Table 2. The onset of puberty (sperm production) occurs at 120–180 days in conventional boars. During the present study, boars were fed ad-lib from 1 to 18 weeks, and subsequently fed restricted diets. Boars were housed individually at an ambient temperature between 18 and 22 °C, and had free access to water.

**Table 1:** Composition of the control diets

Items	15–40 kg	40–60 kg	60–80 kg	80–100 kg	100–130 kg
Corn	65.07	67.06	67.37	70.17	70.14
Soybean meal	20.00	20.00	23.50	21.00	21.50
Wheat bran	3.00	3.00	4.00	4.00	4.00
Fish meal	4.50	3.50	1.00	1.00	1.00
Yeast protein	3.00	2.50	0.00	0.00	0.00
L-lysine	0.46	0.37	0.38	0.29	0.12
D-Methionine	0.07	0.05	0.05	0.02	0.00
L-Threonine	0.15	0.08	0.14	0.11	0.04
Calcium carbonate	0.29	0.33	0.35	0.38	0.41
Dicalcium phosphate	2.05	1.90	2.05	1.87	1.63
Chloride choline	0.15	0.15	0.15	0.15	0.15
Salt	0.25	0.25	0.20	0.20	0.20
Saleratus	0.20	0.00	0.00	0.00	0.00
Vitamin premix^a^	0.21	0.21	0.21	0.21	0.21
Mineral premix^b^	0.60	0.60	0.60	0.60	0.60
Total	100	100	100	100	100

^a^ Vitamin premix are changed during the boars growth according to the NRC(2012), and vitamin E is 100 IU/kg VE as dl-α-tocopheryl acetate in all diets

^b^ Mineral premix are changed during the boars growth according to the NRC(2012)

**Table 2:** Fatty acid composition in diets ^a^

Fatty acid	C	T1	T2	T3
Fish Oil (%)	0	0.2	2	3
Sunflower oil (%)	0	2.8	1	0
Myristic (C14:0)	0.7	0.6	4.7	7.0
Palmitic (C16:0)	13.2	7.2	14.9	19.3
Palmitoleic (C16:1)	0.9	0.7	5.2	7.4
Stearic (C18:0)	2.1	4.9	4.2	3.7
Oleic (C18:1)	29.1	28.6	17.9	11.9
Linoleic (C18:2)	49.4	52.1	19.9	1.6
Linolenic (C18:3)	1.7	0.5	1.2	1.7
Eicosanoic (C20:0)	0.6	0.4	0.8	1.0
Gondoic (C20:1)	0.4	0.6	1.9	2.7
Eicosapentaenoic acid (C20:5)	–	0.8	7.2	10.8
Erurcic acid (C22:1)	–	0.8	2.4	3.3
Docosahexaenoic (C22:6)	–	1.3	11.4	17.2
Other fatty acids	1.9	1.0	7.9	11.9
Total n-3 PUFA	1.7	2.6	19.9	29.6
Total n-6 PUFA	49.4	52.1	19.9	1.7
Ratio of n-6/n-3	29: 1	20:1	1:1	1:18

^**a**^ C, the control group; T1, the 0.2 % fish oil group; T2, the 2 % fish oil group; T3, the 3 % fish oil group. Data expressed as a percentage of total fat acid on the weight basis. Values are means (*n* = 4). “–”: at or below 0.5 % of total fatty acids undetected

### Testicular index measurement

Boars were weighed at 4, 10, and 18 weeks, and then the length and width of boar testis were measured. The testicular longitudinal maximum length and horizontal maximum width between two endpoints were measured using a vernier caliper. Testicular volume was calculated using the formula [19]:

$$ \mathrm{volume} = \frac{4}{3}\uppi \times \kern0.5em \left(\frac{1}{2}\mathrm{length}\right)\times \kern0.5em {\left(\frac{1}{2}\mathrm{width}\right)}^2\times 2 $$

Testis index was expressed as the percentage of average boar testicular volume divided by body weight (kg).

### Lipid analysis in diet and spermatozoa

The fatty acid compositions of diets (oils and diet) were evaluated using Shimadzu GC-14B gas chromatography apparatus (Shimadzu Co., Ltd., Kyoto, Japan) in the Food Quality Testing Center (Cheng du, Sichuan, China) [20]. The fatty acid compositions of the spermatozoa were evaluated by HP6890 GC-FID gas chromatography apparatus (Agilent Technologies, Palo Alto, USA) in the Analysis and Testing Center of China Agricultural University according to the method described by Am-in et al. [21], and the composition of samples calculated based on the standard curve. Each fatty acid proportion was expressed as a percentage of the total fatty acids.

### Semen collection and analysis

Boars were trained to mount a dummy for semen collection at 7 months age. After 4 weeks training period, semen was collected twice per week for 4 weeks using the gloved hand technique. The ejaculation duration and reaction time were recorded during each collection, and 4 layers of sterile gauze was used to filter the semen. The gel-free volume of semen, sperm motility, pH and sperm viability were evaluated according to the methods of the world health organization (WHO) and those described by Kaeoket et al. [22, 23]. More than 200 spermatozoa and 3 fields under 10x microscope magnification (Olympus, Japan) were assessed for evaluation of sperm motility. Moreover, the sportive and linear motion sperm in each field were recorded using a counter. Sperm density, expressed as the number of spermatozoa × 10^8^ cells/ml, was determined using a Makler counting chamber (Sefi-Medical Instruments, Haifa, Israel) [24] for each ejaculate. Sperm abnormalities included those classified as head shape, head size and head number anomalies, as well as tail shape, tail length and tail number anomalies. The gentian violet alcohol solution staining method was used with sperm smear staining, and a minimum of 3 replicates of 100 spermatozoa were counted at 200x magnification using light microscopes and expressed as the sperm deformity rate [11].

Semen samples of each boar collected during the 2^nd^ and 4^th^ week following the training period were immediately centrifuged (Biofuge Heraeus Primor, USA) at 1000 *g* for 15 min. Seminal plasma was stored at −20 °C for future analysis, and semen was gently washed with phosphate buffered saline (PBS), and stored in 200 μl tubes at −80 °C for fatty acid and antioxidant capacity analyses.

### Blood sample collection and hormone assay

Blood for serum samples was collected during the 2^nd^ and 24^th^ week of age by venipuncture of the jugular vein, and centrifuged for 15 min at 4 °C and 3500 *g*/min. Serum was stored at −20 °C for hormone analysis. The concentrations of testosterone (T) and prostaglandins E_2_ (PGE2) were determined using the an ELISA kit (Immunotech, R&D, USA). Absorbance was measured at 450 nm using an automatic enzyme standard instrument (Thermo Electron Corporation, Varioskan™, Waltham, MA, USA). A standard curve was constructed based on the absorbance of standard samples, and each sample hormone concentration was calculated and expressed as ng/ml.

### Anti-oxidant capacity measurement

The total superoxide dismutase (T-SOD), glutathione peroxidase (GSH-Px) activity and total anti-oxidative capacity (T-AOC) of spermatozoa were determined using a commercially available kit (Jiancheng Bioengineering Institute, Nanjing, China), following the manufacturer's protocols. Before the assays, the sperm was homogenized by ultrasonic disintegrator (JY96-II, Shanghai, China) in PBS solution and centrifuged at 1000 *g* for 10 min at 4 °C. The supernatant was used for the analysis. One unit of T-SOD activity was defined as the amount of sample protein capable of inhibiting the reduction of nitro blue tetrazolium (NBT) by 50 % of maximum inhibition, whereby 1 unit of GSH-Px was defined as the amount of enzyme necessary to oxidize 1 μmol/NADPH/min at pH 7.0 at 25 °C. The T-AOC reflects the enzyme and non-enzyme original antioxidant, which can reduce the ferricion (Fe^3+^) to ferrousion (Fe^2+^); the latter combines with phenanthroline to produce a stable chelate, which can be measured by spectrophotography at 520 nm wavelength. One unit of T-AOC was defined as per milligram of tissue protein increasing 0.01 absorbance in 1 min [25]. Malondialdehyde (MDA) concentrations were quantified using the thiobarbituric acid method, which is based on the reaction of MDA with thiobarbituric acid to form a pink chromogen [26], and MDA concentration was expressed as nmol/mg protein.

### Analysis of fructose, acid phosphatase and alpha-glucosidase in seminal plasma

Seminal plasma fructose concentration was measured using the resorcinol colorimetric method [27, 28]. Absorbance values were read at 520 nm against blanks by UV-1100 spectrophotometer (Meipuda instrument Co., Shanghai, China). A set of fructose standards was prepared and used to generate a standard curve. Seminal plasma acid phosphatase (ACP) was determined using the benzene disodium phosphate method [29], and 1 unit of activity was defined as 100 ml of sample reacting with substrate for 30 min at 37 °C to produce 1 mg phenol. Alpha-glucosidase was determined using the glucose oxidase method [30], whereby absorbance was quantified at 505 nm wave length and 1 unit of activity was defined as per ml sample producing 1 μmol of D-glucose /min at 37 °C, pH 6.8.

### RNA extraction and PCR assay

The total ribose nucleic acid (RNA) of samples was extracted using the Invitrogen trizol reagent kit (Invitrogen, Carlsbad, CA, USA). Spermatozoa were thawed in ice water and mixed with 0.25 ml Rnase-free water in a 1.5 ml tube. 0.75 ml trizol solution was then added to the tube, and the sample was violently oscillated at 25 °C in a water bath for 5 min [31]. The remainder of the method was performed according to the manufacturer’s instructions. Reverse transcription was carried out with a commercial kit (Takara prime script™ RT reagent kit with gDNA eraser, Dalian, China) using a conventional PCR machine (Bio-Rad Peltier Thermal Cycler). Primer design and synthesis of genes (Table 3) were performed by Shanghai Biological Engineering Company, China. Gene expression was determined by fluorescence quantitative reagent (SYBR Premix Ex Taq^II^, Takara) using fluorescence quantitative PCR (Bio-Rad CFX96 Real-Time system c1000 Thermal Cycler). All procedures were carried out following the manufacturer’s instructions, and the relative expression levels of genes were normalized with the housekeeping gene, β-actin.

**Table 3:** The primer sequences of target genes and house-keeping gene

Gene	Primer sequences (5’-3’)	Product size (bp)	Gene Bank no.
FAD6	F: GCATCATGCCTACACCAACG	150	NM_001244792
	R: TCCACCTCTCTCAGCCGCTC		
CPT-1	F: ATAGAGACTTCCCTGAGCTGCG	102	AF288789.1
	R: TCTGCATTTCTTGATCAGCCCT		
SOD-1	F: GGCAGAGGTGGAAATGAAGAA	107	GU944822
	R: CAGACCATGGCATGAGGGAAT		
GPx-4	F: GTGTGGTGAAGCGGTACGGT	124	NM_214407
	R: CAGGTGGAAGGCTCTGAGGG		
PPAR-α	F: ATAATGCAATTCGATTTGGGC	115	AF175309.1
	R: AGAGACTTGAGATCTGCGGTCTC		
CYP11A1	F: GAGTAGCAGTGGTAGGGGCAG	159	NM_214427.1
	R: CGAGGGGTTTTAGTGGAGATG		
GADPH	F: CTACAGCAACAGGGTGGTGGA	181	NM_001206359.1
	R: TGGGATGGAAACTGGAAGTCA		

### Statistical analysis

Data were analyzed using SPSS19.0 statistic software and expressed as mean ± standard deviation (SD). Data from the study was examined using analysis of variance (ANOVA). Multiple comparisons by Duncan analysis were used to examine statistical differences among treatments. Statistical significance was taken at the 5 % level.

## Results

### Growth performance

**Fig. 1:**
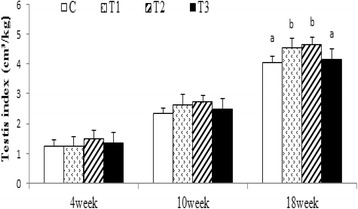
Effects of different ratios of n-6/n-3 PUFA on the testicular volume index of boars (*n* = 8). Testis index = testis volume (cm^3^)/body weight (kg). Values with different letters indicate a significant difference (*P* <0.05)

The feed intake and weight gain of boars are summarized in Table 4. There were no significant differences in bodyweight between boars at the beginning of the experiment, nor in average body weight gain or food intake. However, a significant difference in testis index was identified between treatments (Fig. 1), with the testis index of T1 and T2 significantly higher than T3 and the C group (*P* <0.05).

**Table 4:** Effects of long-chain n-3 fatty acids on the feed intake and body weight gain of boars ^a^

Items	C	T1	T2	T3
Initial body weight (kg)	20.9 ± 0.5	20.1 ± 1.2	20.1 ± 1.2	20.0 ± 1.2
Final body weight (kg)	137.8 ± 7.2	136.8 ± 3.4	135.8 ± 3.3	135.0 ± 5.6
Weight gain (kg)	0.74 ± 0.03	0.79 ± 0.01	0.78 ± 0.02	0.79 ± 0.06
Food intake (kg/d)	2.04 ± 0.08	1.93 ± 0.03	1.93 ± 0.02	1.94 ± 0.07

^a^ C, the control group; T1, the 0.2 % fish oil group; T2, the 2 % fish oil group; T3, the 3 % fish oil group. Data were expressed as means ± standard deviation (*n* = 8)

### Fatty acid composition in spermatozoa

The content of n-3 fatty acid in spermatozoa increased with increasing dietary n-3 fatty acid content. The n-6 and n-3 fatty acid content in spermatozoa of the C group was lower than other treatments (Table 5, *P* <0.05). The n-6/n-3 ratio in T3 boars was significantly lower than in C and T1 boars.

**Table 5:** Effects of long-chain n-3 fatty acids on the composition of spermatozoa (%) ^a^

Fatty acids	C	T1	T2	T3
C6:0	1.24 ± 0.15^b^	0.96 ± 0.15^a^	1.05 ± 0.04^ab^	1.23 ± 0.17^b^
C8:0	20.0 ± 0.6^d^	15.2 ± 0.4^a^	16.6 ± 0.3^b^	18.1 ± 0.6^c^
C10:0	0.54 ± 0.06^b^	0.38 ± 0.05^a^	0.38 ± 0.06^a^	0.52 ± 0.05^b^
C12:0	0.68 ± 0.03^c^	0.49 ± 0.09^ab^	0.38 ± 0.09^a^	0.52 ± 0.04^b^
C14:0	7.43 ± 0.22	7.81 ± 0.41	7.43 ± 0.25	7.34 ± 0.51
C16:0	21.4 ± 0.2^c^	22.9 ± 0.5^d^	19.8 ± 0.5^b^	18.4 ± 0.2^a^
C16:1	0.37 ± 0.04^b^	0.30 ± 0.01^a^	0.38 ± 0.04^b^	0.36 ± 0.02^ab^
C18:0	8.55 ± 0.29^b^	10.02 ± 0.30^c^	8.37 ± 0.34^b^	7.27 ± 0.18^a^
C18:1	1.55 ± 0.06^b^	1.70 ± 0.11^b^	1.55 ± 0.14^b^	1.37 ± 0.07^a^
C18:2n-6	2.44 ± 0.15^b^	2.95 ± 0.22^d^	2.70 ± 0.05^c^	2.21 ± 0.07^a^
C20:0	1.32 ± 0.42^ab^	1.34 ± 0.04^ab^	1.67 ± 0.15^b^	1.02 ± 0.05^a^
C20:3n-6	1.01 ± 0.01^a^	1.32 ± 0.09^b^	1.11 ± 0.25^b^	0.82 ± 0.03^a^
C20:4n-6	2.61 ± 0.12^b^	2.79 ± 0.07^b^	2.65 ± 0.23^b^	2.35 ± 0.16^a^
C22:6n-3	29.2 ± 1.2^a^	32.8 ± 1.5^b^	36.0 ± 0.4^c^	37.1 ± 0.9^c^
n-6 PUFA	6.01 ± 0.33^b^	7.36 ± 0.50^c^	6.50 ± 0.43^b^	5.35 ± 0.19^a^
n-3 PUFA	29.2 ± 1.2^a^	32.8 ± 1.5^b^	36.0 ± 0.4^c^	37.1 ± 0.9^c^
Total n-6/n-3	0.21 ± 0.01^c^	0.23 ± 0.01^d^	0.17 ± 0.01^b^	0.14 ± 0.01^a^

^a^ C, the control group; T1, the 0.2 % fish oil group; T2, the 2 % fish oil group; T3, the 3 % fish oil group. Data expressed as a percentage of total fat acid on the weight basis, and data represent means ± standard deviation (*n* = 6), and values in the same row with different superscript letter means significant difference (*P* <0.05)

### Semen quality

As shown in Table 6, the reaction time (ejaculation time) in T2 boars was significantly shorter than other treatment groups (*P* <0.05). The semen volume of T3 boars was greater than other treatments (*P* <0.05), with no significant differences identified between C, T1 and T2 treatments. Sperm density and the total sperm number per ejaculation in T2 boars were significantly higher than in other treatments (*P* <0.05), and sperm deformity rate was significantly lower (*P* <0.05). However, no significant difference in semen pH, sperm motility and linear motion rate of sperm were identified between treatments.

**Table 6:** Effects of long-chain n-3 fatty acids on the semen quality of boars ^a^

Items	C	T1	T2	T3
Reaction time (s)	25.5 ± 6.6^b^	14.9 ± 3.1^a^	7.4 ± 2.3^a^	8.4 ± 1.8^a^
Persistent period (s)	295.2 ± 52.9	293.0 ± 33.3	280.2 ± 19.9	323.4 ± 74.5
Semen volume (ml)	166.4 ± 34.9^a^	163.3 ± 13.6^a^	201.6 ± 18.9^ab^	219.9 ± 51.7^b^
Sperm motility (%)	76.1 ± 1.9	76.8 ± 2.9	77.5 ± 4.6	72.5 ± 3.6
Sperm viability (%)	79.6 ± 4.5	82.2 ± 2.4	80.2 ± 6.3	78.7 ± 3.7
Sperm density (×10^8^ spz/ml)	2.31 ± 0.24^a^	2.80 ± 0.18^b^	2.96 ± 0.23^b^	2.20 ± 0.48^a^
Deformity ratio (%)	8.64 ± 0.98^c^	4.39 ± 0.74^a^	3.65 ± 0.73^a^	6.34 ± 1.10^b^
pH	6.42 ± 0.21	6.55 ± 0.12	6.53 ± 0.36	6.68 ± 0.44
Sperm number of each ejaculate each ejaculation	346.6 ± 67.8^a^	416.5 ± 51.2^a^	539.3 ± 25.3^b^	423.4 ± 32.6^a^

^a^ C, the control group; T1, the 0.2 % fish oil group; T2, the 2 % fish oil group; T3, the 3 % fish oil group. Data were expressed as means ± standard deviation (*n* = 8), and means on the same row with different superscripts were different significantly (*P* <0.05)

### Seminal plasma biochemistry

Seminal plasma ACP and fructose concentration in the T2 treatment were significantly higher than all other treatments (Table 7, *P* <0.05). However, alpha-glucosidase activity decreased with increasing dietary n-3 PUFA content (*P* <0.05). No obvious difference in MDA level and T-SOD activity was apparent in seminal plasma and sperm between treatments (Table 8). GSH-Px activity of seminal plasma in the T3 treatment was significantly higher than in the C treatment (*P* <0.05), while sperm GSH-Px activity in the T2 treatment was significantly higher than in the T1 treatment (*P* <0.05). After dietary supplementation with oil, seminal plasma T-AOC increased, with no significant difference apparent between T1, T2 and T3 treatments. Dietary n-6/n-3 ratios had no significant effect on sperm T-AOC.

**Table 7:** Effects of long-chain n-3 fatty acids on seminal plasma biochemical of boars^a^

Items	C	T1	T2	T3
Acid phosphatase (U/100 ml)	157.9 ± 16.4 ^a^	273.0 ± 20.1 ^b^	368.7 ± 8.8 ^c^	159.1 ± 14.2^a^
Fructose (g/L)	0.77 ± 0.09 ^b^	0.59 ± 0.06 ^a^	1.14 ± 0.08 ^c^	0.53 ± 0.05^a^
α-Glucosidase (U/ml)	19.7 ± 1.3 ^ab^	21.9 ± 2.3^b^	20.2 ± 2.1 ^ab^	16.1 ± 1.8^a^

^a^ C, the control group; T1, the 0.2 % fish oil group; T2, the 2 % fish oil group; T3, the 3 % fish oil group. Sample were collected from 2^nd^ and 4^th^ week, and data were the average of two weeks and expressed as means ± standard deviation (*n* = 16), and values on the same row with different superscripts differ significantly (*P* <0.05)

**Table 8:** Effects of long-chain n-3 fatty acids on the semen antioxidant capacity of boars^a^

Items	C	T1	T2	T3
MDA				
Seminal plasma (nmol/ml)	1.67 ± 0.25	1.91 ± 0.34	1.77 ± 0.31	1.84 ± 0.15
Sperm (nmol/mg prot)	3.40 ± 0.32	3.56 ± 0.38	3.38 ± 0.38	3.38 ± 0.22
T-SOD				
Seminal plasma (U/ml)	21.3 ± 0.6	21.3 ± 2.2	24.4 ± 2.5	23.1 ± 4.1
Sperm (U/mg prot)	17.8 ± 2.8	26.0 ± 4.1	24.6 ± 2.5	20.7 ± 3.3
GSH-Px				
Seminal plasma (U/ml)	131.9 ± 11.0^a^	139.0 ± 11.6^ab^	144.5 ± 17.3^ab^	158.1 ± 12.6^b^
Sperm (U/mg prot)	18.5 ± 1.7^ab^	16.1 ± 3.6^a^	24.2 ± 1.9^b^	18.1 ± 3.8^ab^
T-AOC				
Seminal plasma (U/ml)	0.64 ± 0.17^a^	0.87 ± 0.16^b^	0.79 ± 0.09^ab^	0.84 ± 0.11^ab^
Sperm (U/mg prot)	5.51 ± 0.99	5.91 ± 0.84	5.69 ± 0.98	5.47 ± 0.94

^a^ C, the control group; T1, the 0.2 % fish oil group; T2, the 2 % fish oil group; T3, the 3 % fish oil group. Sample were collected from 2^nd^ and 4^th^ week, and data were the average of two weeks and expressed as means ± standard deviation (*n* = 16), and values within the same row with different superscripts were significantly different (*P* <0.05). MDA, malondialdehyde; T-SOD, total superoxide dismutase; GSH-Px, glutathione peroxidase; T-AOC, total ant-oxidative capacity

### Serum hormone

Compared to the C group, serum testosterone (T) and PGE_2_ levels were significantly higher in all oil-supplemented treatments (Table 9), with no significant difference for in T between them. Interestingly, PGE_2_ concentration in the T2 treatment was higher than other groups (*P* <0.05), but no significant difference was observed between T1 and T3 treatments.

**Table 9:** Effects of long-chain n-3 fatty acids on the serum hormone levels of boars^a^

Items	C	T1	T2	T3
Testostrone				
2^nd^ week (ng /ml)	7.4 ± 1.5^a^	8.7 ± 1.3^ab^	10.3 ± 1.7^b^	8.5 ± 1.1^ab^
24^th^ week (ng /ml)	7.9 ± 1.7^a^	13.8 ± 1.8^b^	15.8 ± 2.1^b^	13.4 ± 2.1^b^
PGE_2_				
2 ^nd^ week (ng /ml)	188.9 ± 29.5 ^a^	234.7 ± 21.4 ^b^	288.4 ± 19.8^c^	253.3 ± 15.6 ^b^
24 ^th^ week (ng /ml)	215.5 ± 27.5^a^	267.2 ± 29.9^b^	386.9 ± 18.8^c^	308.1 ± 10.2^b^

^a^ C, the control group; T1, the 0.2 % fish oil group; T2, the 2 % fish oil group; T3, the 3 % fish oil group. Data were expressed as means ± standard deviation (*n* = 8), and means within the same row with different superscripts differ (*P* <0.05)

### Gene expression

**Fig. 2:**
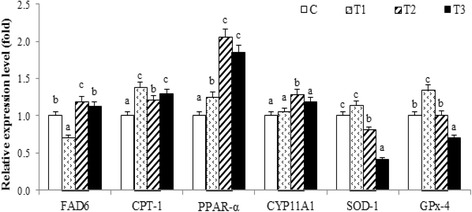
Effects of different ratios of n-6/n-3 PUFA on the expression of sperm functional genes (*n* = 4). Values with different letters indicate a significant difference (*P* <0.05). FAD6: Δ^6^-Fatty acid desaturase; CPT-1: Carnitine palmitoyl transferase-1, SOD-1: Superoxide dismutase-1; GPx-4: Glutathione peroxidase-4; PPAR-α: Peroxidase proliferation activated receptor-α; CYP11A1: Cytochrome P450, family 11, subfamily A, polypeptide 1

Δ6-Fatty acid desaturase (FAD6) and carnitine palmitoyl transferase-1 (CPT-1) are the genes involved in fatty acid metabolism. Results showed significantly higher FAD6 gene expression in the T2 treatment as compared to all other treatments (Fig. 2, *P* <0.05), and significantly increased CPT-1 gene expression in T1 and T3 treatments as compared to the C treatment (*P* <0.05). mRNA levels of SOD-1 and GPx-4 in the T3 treatment, which are related to oxidative stress, were lower than observed for other treatments (*P* <0.05). Furthermore, expression of the peroxidase proliferation activated receptor α (PPAR-α) and cytochrome P450, family 11, subfamily A, polypeptide 1 (CYP11A1) were significantly increased in the T2 treatment as compared to all other treatments (*P* <0.05).

## Discussion

Although there are some reports that fatty acid supplementation may improve the reproduction of human and animal males [9, 32, 33], little research has been done to study the effects of different dietary n-6/n-3 fatty acid ratio before sexual maturity on male reproduction and semen quality. Our data showed that there were no effects on growth performance of breeding boars when they were fed with different n-6/n-3 ratio diets. However, a 1:1 ratio of n-6 to n-3 in the diet improved the testicular development of boars. External testis dimensions, body weight and sperm characteristics are biomarkers of the reproductive performance in of boars [24]; boars with greater testicular volume may have better libido [34], and the testicular size is the key principle for breeding selection [35].

Semen quality was also significantly affected by the dietary n-6/n-3 PUFA ratio during the present study. It has previously been reported that diets with a n-6/n-3 fatty acid ratio of 1.6:1 could increase the proportion of intact acrosome of the boar’s spermatozoa [17]. Oils that are rich in n-3 PUFA also significantly increased sperm density [36] and sperm number [9]. Previously, we reported that the development of testis and the morphological structure of spermatozoa in rats were better with a dietary n-3/n-6 PUFA ratio of 1.52:1 [18]. It was reported that the ratio of n-6/n-3 in boar sperm and seminal plasma was 2.55:1 and 3.35:1, respectively [37], and that a ratio of 1–4:1 n-6/n-3 fatty acids would be beneficial to humans [38]. Additionally, Blesbois et al. [39] found a dietary n-6/n-3 ratio of 10:3 improved the hatch rate of male turkeys. Interestingly, we found that both a high or low dietary n-3/n-6 ratio had adverse effects on sperm motility. As reported by Daraji [40], higher dietary n-6 PUFA (n-6/n-3 = 42.94:1) decreased sperm concentration and the number of normal sperm. Blank et al. [41] reported that ratios of LA:ALA (Linoleic acid: Alpha linolenic acid) lower than 4:1 would have little beneficial effect on docosahexaenoic acid status, suggesting that the proper ratio of dietary n-6/n-3 is important.

It has previously been reported that the composition of sperm fatty acids varies with diet, the content of fatty acids in spermatozoa reflects dietary n-6/n-3 fatty acid ratios, and that the different proportion and source of fatty acids are related to sperm cell membrane structure, lipid composition and acrosome fertilization ability [3, 4, 7]. In fact, PUFA can permeate into sperm cell membranes, significantly affecting the scalability of the sperm plasma membrane and enhancing the osmotic resistance of the acrosomal membrane [10]. Moreover, compared with other oils, the PUFA of fish oil is able to significantly affect the proportion of EPA and DHA in adult boar testicles, and significantly alter the fatty acid composition of testis [16]. Such results further support the conclusion that fatty acid composition of spermatozoa is related to dietary fatty acid composition.

Spermatogenesis and metabolism are regulated by hormones [5]. It is known that testosterone and prostaglandin are associated with fatty acids, and arachidonic acid is usually a precursor of prostaglandin synthesis [5, 7]. Unfortunately, there are few reports of the regulation of hormone synthesis by different PUFA ratios. Hormones regulate the development of the reproductive organs and spermatogenesis. Higher serum testosterone and PGE2 concentrations in the T2 treatment during the present study may indicate improved testis development and superior sperm quality. As reported by Castellano [16], fish oils rich in DHA increased the serum testosterone and estradiol levels in boars. For the rat, it was also demonstrated that dietary fatty acid supplementation altered blood steroid levels [42], and that serum hormone level is related to sperm concentration, motility and morphology [43–45]. We therefore speculate that improvement of sperm quality may be regulated by hormone synthesis and secretion associated with different fatty acid compositions in the diet.

Epididymis and accessory sex glands also play an important role in regulating sperm maturation and fertilization capability [27]. In this study, higher ACP and fructose levels were observed in T2 boars, but higher levels of n-3 or n-6 fatty acid reduced the fructose concentration and alpha-glucosidase activity in the seminal plasma. Currently, few reports exist concerning the n-3/n-6 PUFA regulation of seminal biochemical markers. Biochemical parameters in semen are directly regulated by hormone and proteins in seminal plasma [42, 46]. Theoretically, alteration of secretions from the accessory sex gland may modify the environment of sperm, and subsequently affect sperm quality [47]. ACP are involved in the metabolism of spermatozoa via the hydrolysis of carbohydrates [48] and are associated with semen concentration [49]. Fructose is thought to be a major energy source for sperm movement, and low level of seminal fructose coincided with decreasing sperm motility and fertilization ability [50]. It has been reported that seminal plasma alpha-glucosidase activity reflects the functional state of the epididymis and has an important role in azoospermia [29, 30, 51].

One of the most important factors contributing to poor semen quality has been reported to be oxidative stress [31]. Reports showed that oxidative stress is associated with fatty acid oxidation, which directly affects the survival of sperm [52, 53]. In the current study, it was found that gene expression related to fatty acid metabolism in spermatozoa was altered by changes in dietary n-6/n-3 ratios. Specifically, the mRNA expression of FAD6, CPT-1, PPAR-α and CYP11A1 were up-regulated in T2 boars, which may be associated with sperm fatty acid metabolism or antioxidant capacity. It is well known that PUFA can improve the antioxidant capacity of sperm [10, 54], which may further be related to a change of liver fatty acid composition and antioxidant capacity [52]. However, higher linoleic acid and arachidonic acid can cause the cascade reaction of lipid peroxidation and DNA damage of spermatozoa [53]. Lipid peroxides can be decomposed into MDA, and subsequently inhibit sperm mitochondrial function and enzyme activity, and affect sperm motility [54]. The present study showed that the mRNA levels of GPx-4 and SOD-1 were lower, and the GSH-Px enzyme activity in T2 treatment boars was higher, as compared to the C group. Therefore, an n-6/n-3 ratio of 1:1 may more effectively reduce exogenous oxidative damage in boar sperm, providing a more favorable environment for sperm survival. In contrast, a higher n-3/n-6 ratio is not recommended in the diet of boars.

## Conclusion

In summary, proper n-6/n-3 fatty acid ratio in the diet of breeding boars enhanced the development of testis and accessory sex gland function, and improved sperm quality, which may be related to favorable hormone metabolism and antioxidant capacity. These findings suggest that a balanced dietary n-6/n-3 PUFA ratio before sexual maturity plays an important role in breeding boars, and may also apply to humans and other species.

## Abbreviations

ACP: acid phosphatase
CPT-1: carnitine palmitoyl transferase-1
CYP11A1: cytochrome P450: family 11, subfamily A, polypeptide 1
FAD6: Δ^6^-fatty acid desaturase
GSH-Px: glutathione peroxidase
MDA: malonaldehyde
PGE_2_: prostaglandin E2
PPAR-α: peroxidase proliferation activated receptor-alpha
PUFA: polyunsaturated fatty acid(s)
T: testosterone
T-AOC: total-Antioxidant capacity
T-SOD: total superoxide dismutase
